# RAPID CTA versus JLK LVO for large vessel occlusion detection: a pragmatic comparison of performance and common pitfalls

**DOI:** 10.1007/s00234-026-03921-0

**Published:** 2026-03-10

**Authors:** Hee-Jung Ha, Wi-Sun Ryu, Beom Joon Kim, Myung Jae Lee, Sung Hyun Baik, Cheolkyu Jung, Jae Hyoung Kim, Jae W. Song, Sami Al Kasab, Leonard Sunwoo

**Affiliations:** 1 Department of Neurology, Ewha Womans University College of Medicine, Seoul, Republic of Korea; 2 Artificial Intelligence Research Center, JLK Inc., Seoul, Republic of Korea; 3 Department of Neurology, Seoul National University Bundang Hospital, Seongnam-si, Republic of Korea; 4 Department of Radiology, Seoul National University Bundang Hospital, Seongnam-si, Republic of Korea; 5 Department of Radiology, Hospital of the University of Pennsylvania, Philadelphia, PA US; 6 Medical University of South Carolina, Charleston, SC US; 7 Department of Radiology, Seoul National University College of Medicine, Seoul, Republic of Korea

**Keywords:** Acute ischemic stroke, Large vessel occlusion, Computed tomography angiography, Automated software, Deep learning

## Abstract

**Purpose:**

Rapid detection of large vessel occlusion (LVO) is crucial for improving outcomes of acute ischemic stroke. This study provides a real-world, head-to-head comparison of two commercial AI tools for automated LVO detection—RAPID CTA (vessel density-based) and JLK LVO (deep learning-based)—in a Korean stroke center.

**Methods:**

We retrospectively analyzed 176 consecutive patients with suspected stroke who underwent both CT angiography and CT perfusion. The performance of RAPID CTA and JLK LVO was compared against expert neuroradiologist consensus using the area under the receiver operating characteristic curve (AUROC). Misclassified cases (false positives [FPs] and false negatives [FNs]) were reviewed to determine their underlying causes.

**Results:**

LVO was confirmed in 53 of 176 patients (30.1%). Both tools demonstrated high and comparable overall performance (AUROC 0.93 for both, *p* = 0.64). The causes for misclassifications were also consistent across both platforms. The most common cause of FPs was high-grade intracranial stenosis mimicking occlusion. The primary cause for FNs was the presence of well-developed collateral flow in distal occlusions, which masks the vessel cut-off. However, a matched-sensitivity analysis revealed different performance trade-offs; at a predefined threshold yielding 83% sensitivity, JLK LVO demonstrated higher specificity than RAPID CTA (0.96 vs. 0.89).

**Conclusion:**

Both RAPID CTA and JLK LVO are effective tools, but they exhibit distinct performance trade-offs. A clear understanding of each tool’s common pitfalls and performance trade-offs is crucial for clinicians to effectively integrate these AI results for optimal patient care.

**Supplementary Information:**

The online version contains supplementary material available at 10.1007/s00234-026-03921-0.

## Introduction

Large vessel occlusion (LVO) is a major cause of severe neurological deficits and poor prognosis in patients with acute ischemic stroke [1]. Therefore, rapid detection of LVO and timely reperfusion therapy are crucial for the functional recovery of patients [2, 3]. As clinical trials have successfully expanded the time window for treatment, a greater number of patients are now eligible for these interventions [4, 5]. This has placed a growing burden on medical professionals in emergency settings to rapidly diagnose LVO with exceptional speed and accuracy. This challenge is magnified in urgent situations or in hospitals without immediate access to expert radiologists, where computed tomography angiography (CTA), the standard diagnostic tool, must be interpreted swiftly and accurately.

Artificial intelligence (AI) has emerged as a promising solution to this diagnostic challenge, with several commercial software tools now available for automated LVO detection on CTA scans [6]. However, the performance of these AI tools in the controlled environment may not translate directly to routine clinical practice. The diagnostic accuracy can be influenced by variables like disease prevalence and the specific patient population, leading to concerns about the generalizability of their study results [7]. Furthermore, direct head-to-head comparison studies between commercial software tools, which could provide valuable information to clinicians, are scarce [8, 9]. Furthermore, such studies are particularly rare in Asian populations, where LVO etiology can differ from Western populations [10].

For any AI diagnostic tool to be trusted in clinical practice, its accuracy must be considered alongside a clear understanding of its errors [11]. In LVO detection, where a diagnostic error can lead to devastating patient outcomes, a detailed analysis of such cases is essential. Providing this level of analysis is fundamental to building clinical confidence and ensuring that AI tools are used safely and effectively.

This study was designed to address these issues through a direct, head-to-head comparison of two commercial LVO detection software products, RAPID CTA (RapidAI, Menlo Park, CA) and JLK LVO (JLK Inc., Seoul, Republic of Korea). We evaluated these tools using CTA images from all consecutive patients, including both stroke and non-stroke cases, who presented to the emergency department of a single tertiary stroke center. In addition to comparing overall performance, we conducted a detailed analysis of the false positive (FP) and false negative (FN) cases from each software. Through this evaluation, we aimed to provide clinicians with practical insights to better inform their use of these emerging AI technologies.

## Methods

Data supporting the findings of this study are available from the corresponding author upon reasonable request from qualified researchers, subject to privacy and ethical restrictions. This study was conducted in accordance with the Standards for Reporting Diagnostic accuracy studies (STARD).

### Study population

From June 2024 to February 2025, we enrolled consecutive adult patients aged 18 years or older who presented to the emergency department of Seoul National University Bundang Hospital with acute neurological symptoms suspicious for acute ischemic stroke, and who underwent both CTA and CT perfusion (CTP) as part of their initial diagnostic workup. Patients diagnosed with acute ischemic stroke (including those with and without LVO) and non-stroke patients (e.g., diagnosed with stroke mimics or other neurological conditions not involving acute stroke) were included. Patients were excluded if their CTA images could not be successfully processed or analyzed by the software due to technical reasons or the presence of severe motion artifacts. The study protocol was approved by the institutional review board of Seoul National University Bundang Hospital (No. B-2507-987-107) and a written informed consent was waived.

### Data collection

For all enrolled patients, regardless of their final diagnosis, demographic information, including age and sex, was collected, along with the final diagnosis (stroke or non-stroke). For patients diagnosed with stroke, more detailed clinical data, including medical history and comorbidities, National Institutes of Health Stroke Scale (NIHSS) score upon emergency department arrival, and key time metrics, including time of symptom onset and time of CTA acquisition, were collected.

All CTA and CTP examinations were performed using a single, dedicated CT scanner (Brilliance iCT 256, Philips Healthcare, Best, The Netherlands). In our institution, the standard protocol for suspected acute ischemic stroke with LVO involves an initial non-contrast CT (NCCT), followed by CTP and 3-phase multiphase CTA (mCTA). For NCCT, parameters were tube voltage of 120 kVp, tube current of 250 mAs (CTDIvol: 33.4 mGy), and detector collimation of 64 × 0.625 mm. CTP was acquired at 80 kVp, 150 mAs (CTDIvol: 7.4 mGy), and 64 × 1.25 mm, and mCTA at 80 kVp, 170 mAs (CTDIvol: 7.3 mGy), and 128 × 0.625 mm.

A total of 95 mL of Iomeprol 400 (Iomeron^®^; Bracco, Milan, Italy) was injected via the right antecubital vein (50 mL for CTP and 45 mL for mCTA) at a rate of 4–5 mL/s using an automated power injector, followed by a saline flush of 30 mL and 50 mL, respectively. Automated bolus-tracking in the descending aorta triggered CTP at 130 HU. For the subsequent mCTA, a 180 HU threshold was used to account for residual contrast from the preceding CTP phase. The first mCTA phase covered from the carina level to the vertex, while the second and third phases covered from C2 to the vertex. All images were transferred to each software server installed in the hospital for processing and the results were returned and saved into the picture archiving and communication system (PACS).

### LVO assessment and ground truth establishment

The ground truth for the presence and precise location of LVO was established through a consensus diagnosis by two independent, board-certified neuroradiologists (L.S. and S.H.B., each with 15 years of experience), who were blinded to the results generated by both software tools. The readers performed a comprehensive review using thin-section source images, maximum intensity projection (MIP), and thick-slab MIP images. In cases of diagnostic ambiguity, the thin-section source images were considered the definitive reference for identifying endovascular continuity.

To improve diagnostic precision, particularly for distal occlusions (e.g., middle cerebral artery (MCA) M2 segments), the reviewers utilized CTP maps generated by JLK CTP (JLK Inc.). Occlusions were confirmed by identifying corresponding territorial hypoperfusion—specifically areas with Tmax > 6 s and CBF < 30%. This geographic footprint helped differentiate true occlusions from slow-filling vessels supported by robust collaterals, particularly when perfusion deficits showed partial MCA territory involvement with sparing of the basal ganglia, characteristic of M2 occlusions.

LVO was defined as a sharp cut-off of contrast opacification on CTA in the anterior circulation, including intracranial internal carotid artery (ICA) (i.e., from petrous to terminal ICA or combined ICA and middle cerebral artery (MCA) occlusions), and MCA (M1 or M2 segments). Other arterial occlusions were excluded as they fall outside the intended diagnostic scope of the software tools evaluated.

### Software analysis

CTA image datasets were independently processed by two commercial software platforms: RAPID CTA (version 5.0(g)) and JLK LVO (version 3.2.0.3). To maintain consistency with the software’s intended purpose, only the first phase of the mCTA—representing peak arterial opacification—was utilized for automated analysis by both RAPID CTA and JLK LVO.

RAPID CTA utilizes a rule-based algorithm that identifies LVO by comparing vessel density and opacification between hemispheres. Its output is presented as a percentage reduction in vessel density compared to the contralateral side, categorized into discrete ranges: 0–45%, 45–60%, 60–75%, 75–80%, and normal.

JLK LVO is a deep learning-based tool developed on a multicenter dataset of 2,045 patients [12]. The algorithm architecture consists of three core components: automated MIP image generation, vessel segmentation using a 2D U-Net with Inception modules, and occlusion classification via EfficientNetV2. Its performance has been established in multiple validation studies showing high AUCs ranging from 0.94 to 0.97 [13, 14]. The tool generates a probability score (ranging from 0 to 1) indicating the likelihood of LVO presence, where a higher score signifies a greater likelihood of occlusion.

### Diagnostic performance evaluation

The diagnostic performance of each software was evaluated against the expert-adjudicated ground truth. Given that JLK LVO provides a continuous probability score and RAPID CTA provides categorized results, we calculated the area under the receiver operating characteristic curve (AUROC) as the primary metric for comparing the overall discriminatory ability of the two software tools. For a more detailed assessment of clinical utility, secondary diagnostic performance metrics included sensitivity, specificity, positive predictive value (PPV), negative predictive value (NPV), and overall accuracy. For JLK LVO, we used a predefined probability threshold of 0.5 to dichotomize outputs, with probability values ≥ 0.5 considered positive for LVO. For RAPID CTA, LVO was defined by a hemispheric asymmetry that resulted in a 60% or greater reduction in the total contrast-filled vessel volume (i.e. vessel density) compared to the contralateral side, corresponding to the categories of 45–60% or 0–45% [9]. This threshold selection was based on prior literature demonstrating optimal diagnostic performance at this cutoff (Youden index maximum at category 2; attenuation reduction of 45–60%) in a large-scale validation study including MCA M2 segment [15].

To further explore the diagnostic implications of threshold selection, we performed matched-sensitivity analyses. As RAPID CTA provides ordinal categorical outputs, we calculated the sensitivity at each possible threshold level (e.g., 0–45%, 0–60%, 0–75%). We then identified the JLK LVO probability threshold that resulted in the same sensitivity and compared the corresponding specificities of both software platforms. This allowed us to evaluate the trade-offs in specificity at fixed sensitivity levels, offering clinically meaningful insight for decision-making under different risk tolerance scenarios.

To analyze sensitivity according to occlusion location, cases were categorized based on the most proximal lesion site: the intracranial ICA, the M1 segment, or the M2 segment of the MCA. Based on this criterion, multiple vessel occlusions involving an intracranial ICA component were included in the ICA group. This stratified analysis aimed to assess each tool’s robustness in detecting both proximal (ICA and MCA M1) and distal (MCA M2) LVOs and to identify location-specific limitations in diagnostic performance.

### Analysis of false positives and false negatives

A detailed qualitative and quantitative analysis of FP and FN cases were performed for each software. An experienced neuroradiologist (L.S.) reviewed these cases to identify common underlying reasons.

### Statistical analysis

Data were presented as median (interquartile range) or frequency (percentage) as appropriate. We calculated diagnostic performance metrics (sensitivity, specificity, PPV, NPV, accuracy, AUROC) with 95% confidence intervals. The AUROCs of the two software tools are compared using DeLong’s test. McNemar’s test was used to compare the paired sensitivities and specificities between RAPID CTA and JLK LVO. All statistical analyses were performed using R statistical software (version 4.4.3; R Foundation for Statistical Computing, Vienna, Austria). A two-sided p-value of less than 0.05 will be considered to indicate statistical significance.

## Results

### Study population characteristics

During the study period from June 2024 to February 2025, a total of 176 patients were enrolled. After review, no patients met the exclusion criteria, resulting in a final cohort of 176 patients for analysis. The median age of the study population was 71 years (interquartile range, 59–81), and 60.8% were male. Among the included patients, 118 (67%) were ultimately diagnosed with acute ischemic stroke, and 13 (7.4%) with transient ischemic attack (Table 1). LVO was confirmed in 53 patients (30.1%). The median time from symptom onset to CTA acquisition was 3.12 h (IQR, 1.60–7.16). For stroke patients, the median NIHSS score at admission was 11 (IQR, 5–18.25). Among the 53 LVO cases, there were 19 isolated ICA occlusions, 18 isolated M1 segment occlusions, and 11 isolated M2 segment occlusions. An additional 5 cases were diagnosed with multiple vessel occlusions, all of which involved the ICA.

**Table 1:** Clinical and imaging characteristics of study population (*N* = 176)

Characteristics	Value
Age, years	71.0 (59.0–81.0)
Initial NIHSS score*	9.0 (5.0–18.0)
Neurological symptom onset to CTA, hours	3.12 (1.60–7.16)
Male	107 (60.8%)
Diabetes	53 (30.1%)
Atrial fibrillation	27 (15.3%)
Hypertension	100 (56.8%)
Dyslipidemia	87 (49.4%)
Final diagnosis	
No stroke	45 (25.6%)
Acute ischemic stroke	118 (67%)
Non-LVO stroke	69 (39.2%)
LVO stroke†	49 (27.8%)
Transient ischemic attack	13 (7.4%)
Anterior circulation large vessel occlusion (*n* = 53)	
Isolated vessel occlusion	48 (90.6% of LVO)
ICA‡	19 (35.8% of LVO)
MCA M1	18 (34.0% of LVO)
MCA M2	11 (22.9% of LVO)
Multiple vessel occlusions§	5 (9.4% of LVO)
ICA + M1	3 (5.7% of LVO)
ICA + M2	2 (3.8% of LVO)

*NIHSS score was available in 172 patients

†Among 53 patients classified as LVO based on CTA, 4 were not finally diagnosed with acute ischemic stroke and were considered to have chronic occlusions

‡Within the ICA occlusion group, two cases were identified as T-type occlusions (terminal ICA involving both MCA and ACA)

**§**Occlusion site classification in subgroup analysis was based on the most proximal intracranial vessel

### Diagnostic performance of software tools

For detecting LVO in CTA, two software platforms yielded similar results with AUROC for RAPID of 0.93 (95% CI, 0.88–0.97) and JLK LVO of 0.93 (95% CI, 0.88–0.99, *p* = 0.64, Table 2 and Supplementary Fig. 1). At the predefined threshold (< 60% for RAPID CTA and > 0.5 for JLK LVO), both platforms achieved identical sensitivity (0.83, Table 2 and Supplementary Fig. 2). Specificity was numerically higher for JLK LVO (0.96) compared to RAPID CTA (0.89). The positive predictive value was 0.90 for JLK LVO and 0.77 for RAPID CTA, and the negative predictive value was 0.93 for both. Overall accuracy was 0.92 for JLK LVO and 0.88 for RAPID CTA. McNemar’s test for binary classification agreement showed no significant difference (*p* = 0.118).

**Table 2:** Overall diagnostic performance of JLK LVO and RAPID CTA in predefined thresholds

Model	RAPID CTA (Threshold: <60%)	JLK LVO (Threshold: 0.5)
AUROC (95% CI)	0.93 (0.88–0.97)	0.93 (0.88–0.99)
p-value ^a^	0.640	
Sensitivity	0.83 (0.72–0.92)	0.83 (0.74–0.92)
Specificity	0.89 (0.84–0.94)	0.96 (0.92–0.99)
PPV	0.77 (0.68–0.87)	0.90 (0.82–0.98)
NPV	0.93 (0.88–0.96)	0.93 (0.89–0.97)
Overall Accuracy	0.88 (0.82–0.92)	0.92 (0.88–0.96)
p-value^b^	0.118	

^a^p-value calculated by DeLong test

^b^p-value calculated by McNemar’s test

*AUROC *area under the receiver operating characteristic curve, *NPV *negative predictive value, *PPV *positive predictive value

### Matched-sensitivity comparison

A sensitivity-matched analysis demonstrated that both platforms achieved comparable performance across various thresholds (Supplementary Table 1). When RAPID CTA’s sensitivity was set at 0.91 (at a < 80% threshold), the corresponding JLK LVO threshold was 0.07, yielding similar specificities of 0.80 for JLK LVO and 0.81 for RAPID CTA. At a matched sensitivity of 0.87 (RAPID CTA threshold < 75%), the JLK LVO threshold was 0.11, resulting in specificities of both 0.85.

Notably, at lower sensitivity settings, JLK LVO demonstrated higher specificity. When sensitivity was matched at 0.83 (RAPID CTA threshold < 60%), the JLK LVO threshold of 0.42 produced a specificity of 0.95, compared to 0.89 for RAPID CTA. This trend continued at a sensitivity of 0.75 (RAPID CTA threshold < 45%), where the JLK LVO threshold of 0.82 yielded a perfect specificity of 1.00, compared to 0.99 for RAPID CTA.

### Sensitivity according to occlusion site and laterality

Sensitivity was compared across different occlusion sites using predefined thresholds (< 60% for RAPID CTA and ≥ 0.5 for JLK LVO). For the 19 ICA occlusions identified (Table 1), both platforms demonstrated a sensitivity of 0.92 (Supplementary Table 2). This included two T-type occlusions (terminal ICA involving both MCA and ACA), both of which were correctly identified by both software tools. For MCA M1 occlusions, the sensitivity was 1.00 for JLK LVO and 0.94 for RAPID CTA. For M2 occlusions, the sensitivity was 0.30 for JLK LVO and 0.40 for RAPID CTA. In cases of bilateral occlusions, the sensitivity was 1.00 for JLK LVO and 0.75 for RAPID CTA (Supplementary Table 3).

### Analysis of false positives and false negatives

**Fig. 1:**
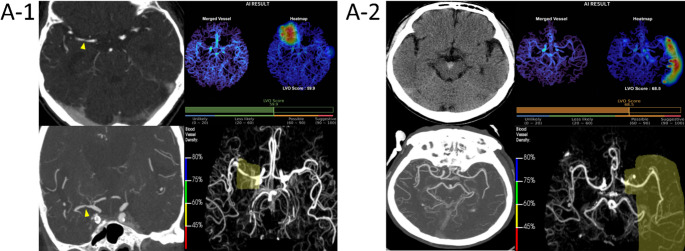

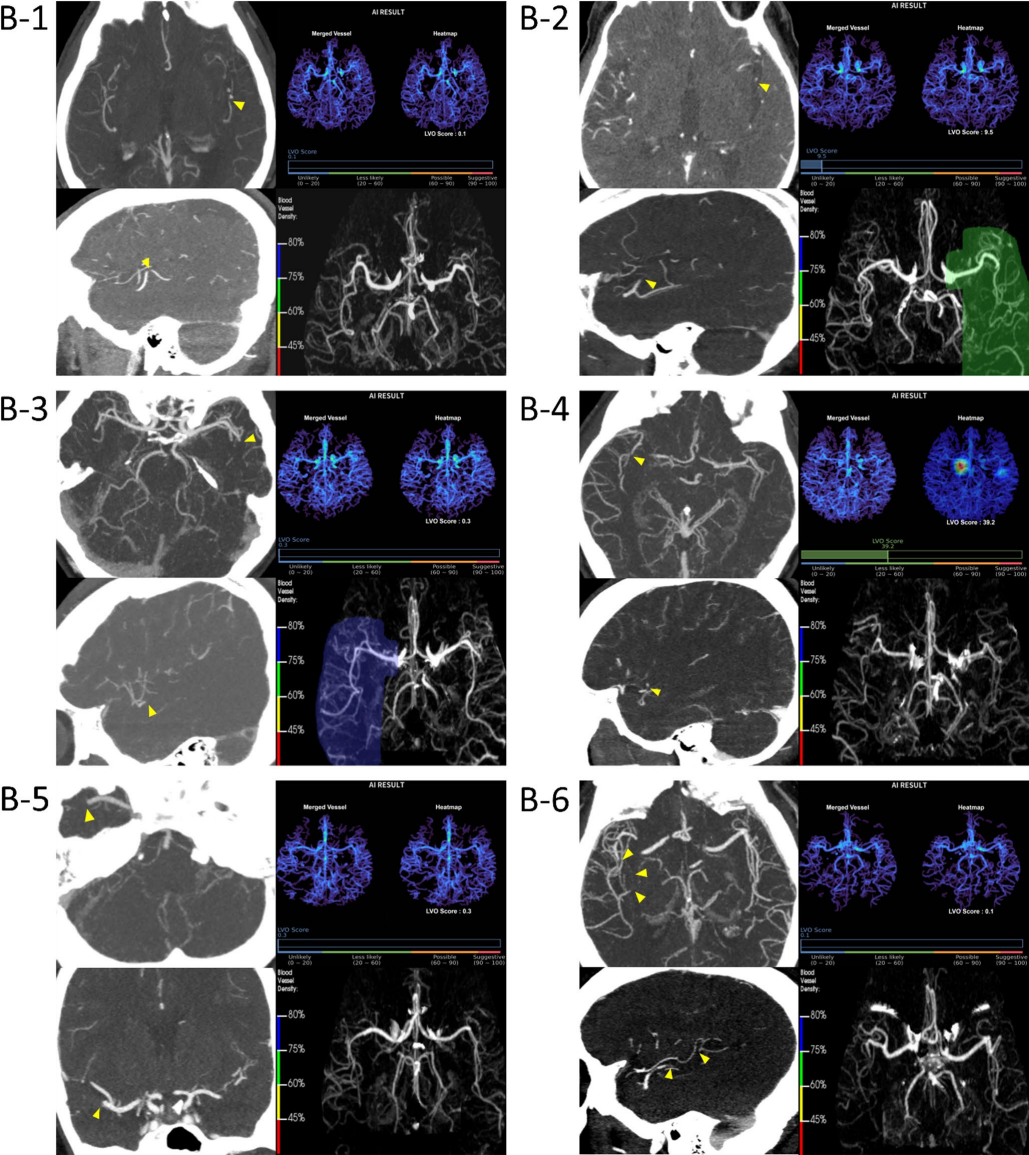
Common False-Positive and False-Negative Cases. (A-1) A 77-year-old male with focal moderate stenosis in the right MCA M1 segment (arrowheads), which was misinterpreted as an occlusion by both algorithms. (A-2) A 63-year-old male presented with a small amount of interpeduncular subarachnoid hemorrhage (arrowheads). There was no evidence of vasospasm or steno-occlusive disease. B-1) An 89-year-old male with a short-segment left MCA M2 occlusion (arrowheads). The finding was likely missed by both algorithms due to the short lesion length and good collateral flow. (B-2) A 71-year-old male with a left MCA M2 inferior division occlusion (arrowheads). The lesion was not detected, likely due to the preserved distal vessel density. (B-3) A 49-year-old male with an occlusion of a distal left MCA M2 branch (arrowheads). The false-negative result was likely due to the lesion's small caliber and the presence of moderate collateral circulation. (B-4) A 56-year-old male with a short-segmental occlusion of right MCA M2 inferior division (arrowheads) accompanied by focally poor collateral flow. The short lesion length likely contributed to the false-negative result. (B-5) A 26-year-old female with a right MCA M2 inferior division occlusion (arrowheads). The false-negative finding was likely attributable to robust collateral circulation. (B-6) An 89-year-old male with a segmental occlusion in the distal right MCA M2 inferior division (arrowheads), accompanied by good collateral flow. CTA= computed tomography angiography, MIP= maximum intensity projection, LVO= large vessel occlusion, MCA= middle cerebral artery

Based on the predefined threshold (< 60% for RAPID CTA and ≥ 0.5 for JLK LVO), a total of 13 FP cases were identified for RAPID CTA and 5 for JLK LVO, with 2 cases being common to both solutions (both cases illustrated in Fig. 1). For FN cases, 9 were identified for each solution, with 6 cases overlapping (all shown in Fig. 1). All erroneous cases were thoroughly reviewed and categorized into groups based on vascular or technical causes (Table 3).

**Table 3:** Potential systematic errors in automated LVO detection

False Positive
**A. LVO mimics**
A-1. High-grade Intracranial Atherosclerotic Disease (ICAD) without occlusion
A-2. Non-atherosclerotic vasculopathies (e.g., cerebral vasospasm, vasculitis, dissection)
A-3. Congenital anatomic variants (e.g., fetal-type PCA, early bifurcation of the M1 segment, hypoplasia)
**B. Technical pitfalls**
B-1. Suboptimal image acquisition or poor contrast opacification
B-2. Intervention related artifacts including coil embolization and intracranial stent insertion
B-3. Algorithm-specific misclassification (e.g., segmentation/localization error, vessel misidentification)
C. Unclassified*
False Negative
**A. Insufficient occlusion signal**
A-1. Occlusion of small caliber or distal vessels (e.g., distal MCA M2 occlusion)
A-2. Short segmental occlusion
**B. Misleading patency signal**
B-1. Hemodynamic compensation by collaterals
B-2. Reconstitution of distal flow
**C. Technical & Anatomic Limitations**
C-1. Artifacts in challenging anatomic locations (e.g., beam hardening artifact at petrous/cavernous ICA)

* Cases with no plausible explanation

*LVO *large vessel occlusion, *ICA *internal carotid artery, *PCA* posterior cerebral artery

For each case, the left panels display the patient’s original scans (CTA source, CTA thick-slab MIP or NCCT), while the right panels show the secondary capture results from JLK LVO (top) and RAPID CTA (bottom).

Panels A-1 and A-2 show FP cases in which both solutions erroneously indicated LVO despite the absence of true occlusion.

Panels B-1 to B-6 show FN cases where both tools failed to detect existing LVOs.

### False positive cases

The possible causes of FP results were classified as either (A) LVO mimics, (B) technical pitfalls, or (C) unclassified (Supplementary Table 4). For the 13 FPs from RAPID CTA, the causes comprised LVO mimics (5 cases, 38.5%), primarily due to high-grade intracranial atherosclerotic disease (ICAD; Fig. 1 A) and technical pitfalls (3 cases, 23.1%). Five cases remained unclassified after expert review (38.5%), In contrast, for the 5 FPs from JLK LVO, technical pitfalls were the predominant cause (3 cases, 60.0%). Two FP cases were common to both tools; one was an LVO mimic and the other was unclassified.

### False negative cases

The most frequent cause for FNs in both solutions was a mixture, combining an ‘insufficient occlusion signal’ (Category A) with a ‘misleading patency signal’ (Category B, Table 5). This pattern, typically a small or segmental occlusion in the presence of good collaterals (Fig. 1B and, B-2, B-5, B-6), accounted for 55.6% of FNs for both RAPID CTA and JLK LVO (5 of 9 cases each). This mixed etiology was also the dominant pattern among the 6 FN cases common to both solutions, accounting for 4 of these cases (66.7%). The remaining common FNs were attributed solely to an insufficient occlusion signal (Supplementary Table 5).

## Discussion

This study compared the diagnostic performance of two automated LVO detection tools, RAPID CTA and JLK LVO, and found that both platforms have high discriminatory ability for detecting LVOs in a South Korean stroke center. This level of performance provides valuable diagnostic support to physicians, especially in time-critical situations or when immediate expert review is not available.

While both tools show comparable overall diagnostic performance as measured by AUROC, their performance is not identical, with notable differences in specificity at clinically relevant sensitivity thresholds. This finding aligns with previous literature for RAPID CTA. A study by Amukotuwa et al. [15] demonstrated this performance variability, reporting that a threshold prioritizing sensitivity (< 75%) achieved 95% sensitivity but only 79% specificity, while another setting (< 60%) improved specificity to 86% at the cost of reducing sensitivity to 91%. In our own analysis, we observed this trade-off directly in our comparison: at high-sensitivity settings commonly used in clinical screening, JLK LVO showed higher specificity, whereas RAPID CTA performed better at thresholds prioritizing specificity. This highlights that the sensitivity-specificity balance is not uniform across the tools, a finding with direct clinical relevance. While high sensitivity is generally favored in acute stroke triage to minimize the risk of missed LVOs, the associated increase in FPs can lead to unnecessary evaluations and resource utilization. In this context, JLK LVO’s ability to maintain higher specificity in high-sensitivity settings offers a potential advantage in reducing FP-triggered downstream interventions. Ultimately, our findings reinforce that optimal threshold selection for each tool must be guided by specific clinical goals and workflow considerations.

Analysis of misclassified cases revealed important insights. Most FP detections were due to LVO mimics like high-grade stenosis or non-atherosclerotic vasculopathies, suggesting current algorithms cannot reliably distinguish true occlusions from hemodynamic changes. In particular, in patients with known ICAD, positive AI results should be interpreted with caution, considering the elevated risk of FP detection. FN cases, in contrast, offer an even more critical perspective. The most common underlying mechanisms involved short segment or distal branch occlusions masked by well-developed collateral circulation—scenarios in which vessel density appears preserved despite true occlusion. These mixed mechanism cases expose an inherent vulnerability in vessel density-based interpretation and suggest that deep learning algorithms trained on such image data may be subject to similar biases.

Our study’s design provides a pragmatic assessment of performance, contrasting with prior studies that exclusively included confirmed stroke patients [13–15]. This approach, which comprised a diverse cohort of both non-stroke and stroke patients, likely explains our findings of lower overall sensitivity but higher specificity. Furthermore, the methodological rigor of our study was enhanced by a meticulous definition of anterior circulation LVO, aligning with the tools’ intended purpose. This contrasts with other reports that included a broader range of occlusions, such as in the basilar artery, which may have inadvertently underestimated the software’s true performance for its designed application [16, 17]. Finally, a critical gap exists in evaluating LVO detection software in Asian populations, where a higher prevalence of ICAD presents a unique diagnostic challenge [18, 19]. Our study, situated in a Korean stroke center, is designed to address this gap.

The similar performance and failure profiles of RAPID CTA, a rule-based density assessment tool, and JLK LVO, a deep-learning model, offer valuable insights into the current state of automated LVO detection. The considerable overlap in FN cases and comparable performance across different occlusion locations suggests that both systems face similar challenges. Specifically, while both tools showed limited sensitivity for distal M2 occlusions, they demonstrated perfect reliability in detecting the most proximal LVOs; both platforms correctly identified 100% of the T-type occlusions present in our cohort. This may indicate that deep learning models could be learning to prioritize imaging features, such as vessel attenuation or loss of opacification, that are also fundamental to traditional rule-based systems. These observations highlight that the primary challenge in automated LVO detection may be less about the specific algorithm and more about the inherent limitations of CTA data itself. Certain occlusion patterns, particularly when influenced by confounding hemodynamic signals, venous contamination or artifacts, remain difficult to reliably detect through computational methods alone [8]. Consequently, integrating AI-driven predictions with other imaging modalities, such as perfusion imaging, non-contrast CT, or MRI, along with a thorough neurological assessment, remains the recommended approach for accurate diagnosis and effective treatment planning.

Furthermore, while our study focuses on the standalone performance of these tools, their primary clinical utility often lies in their role as a decision-support aid to human interpretation. Previous research specifically evaluating the JLK LVO algorithm in an interactive setting demonstrated that AI assistance significantly improves the diagnostic accuracy of early-career physicians [14]. In that study, AI support increased the LVO detection sensitivity of residents by 4.0% and the average AUROC from 0.944 to 0.967 (*P* < 0.001). These findings suggest that even with the algorithmic challenges identified in our current work—such as M2 detection—the synergy between AI and healthcare workers can effectively bridge the expertise gap in time-critical emergency settings, optimizing overall stroke triage workflows.

The evolution of these AI tools must focus on greater clinical nuance and accuracy. A critical advancement will be improving alert specificity by differentiating acute from chronic occlusions, thereby reducing unnecessary activations for stable findings. Furthermore, enhancing detection sensitivity is crucial. This includes both improving algorithmic performance for more distal occlusions, such as in the M2 segment where both tools demonstrated limited performance in our study, and integrating clinical data like symptom laterality to create a more robust and context-aware diagnostic tool.

Our study had several limitations. First, as a single-center study utilizing a single CT vendor, the generalizability of our findings regarding the software tools’ performance in regional stroke centers or with different CT vendors is limited. However, the comparable results with prior studies conducted across diverse populations and with various CT vendors [14, 15] suggest that these software tools may exhibit generally consistent performance. Second, the small sample size for specific locations, particularly MCA-M2 (*n* = 10), limits the statistical power for head-to-head subgroup comparisons. While our cohort reflects a real-world clinical distribution, results for medium vessel occlusion (MeVO) should be interpreted cautiously. Third, although the subjects were consecutively enrolled, the analysis was performed retrospectively. Our study was therefore limited to the standalone performance of the two AI models and did not assess their downstream impact on physician decision-making or final diagnostic accuracy. Therefore, future large-scale prospective studies are warranted to determine the tools’ real-world clinical utility by evaluating their influence on clinician behavior and patient outcomes across diverse settings.

The practical value of this comparative analysis lies in facilitating the transition from ‘AI validation’ to ‘safe clinical implementation’ [20]. Automated LVO tools function most effectively as decision-support systems rather than autonomous diagnostic agents. However, their use carries a risk of ‘automation bias,’ where clinicians may unintentionally over-rely on AI notifications. By delineating that both platforms share specific failure modes—particularly in scenarios involving robust collateral flow or high-grade ICAD mimics—our findings provide clinicians with the necessary context to maintain clinical vigilance. Understanding these performance trade-offs transforms the AI from an opaque ‘black box’ into a transparent assistant. This awareness fosters a more nuanced synergy between computational speed and expert clinical judgment, ensuring that AI-driven triage remains a safe and reliable component of the hyperacute stroke workflow [8, 21].

In conclusion, both JLK LVO and RAPID CTA demonstrate high and comparable diagnostic performance for LVO detection in a real-world clinical setting. While both platforms are highly reliable for proximal occlusions, they are not infallible and show shared challenges in scenarios involving significant collateral circulation or distal occlusions. These findings underscore that automated tools function most effectively as decision-support aids that augment, rather than replace, clinical expertise. Successful clinical integration requires a transition from simple validation to informed implementation, where clinicians are aware of performance trade-offs and synergize AI output with their expert judgment to ensure safe and accurate stroke triage.

## Supplementary Information

Below is the link to the electronic supplementary material.

Supplementary Material 1

## Data Availability

Data supporting the findings of this study are available from the corresponding author upon reasonable request from qualified researchers, subject to privacy and ethical restrictions.
