# Supplementary material for: RAPID CTA versus JLK LVO for large vessel occlusion detection: a pragmatic comparison of performance and common pitfalls
Source: Neuroradiology. 2026 Mar 10;68(4):877–86. doi: 10.1007/s00234-026-03921-0 (PMC13139262; doi:10.1007/s00234-026-03921-0)
Supplement: Supplementary file 1 — Supplementary Material 1 [file 234_2026_3921_MOESM1_ESM.pdf]

Supplementary Materials

RAPID CTA versus JLK LVO for Large Vessel Occlusion Detection: A Pragmatic Comparison of Performance and Common Pitfalls

Supplementary Table 1. Specificity comparison at matched sensitivity levels between JLK LVO and RAPID CTA

RAPID CTA threshold, relative vessel density	RAPID CTA sensitivity at threshold	RAPID CTA Specificity at threshold	JLK LVO specificity at matched sensitivity*
<80%	0.91	0.81	0.80
<75%	0.87	0.85	0.85
<60%	0.83	0.89	0.95
<45%	0.75	0.99	1.00

*For each RAPID CTA relative vessel density threshold (<80%, <75%, <60%, and <45%), the JLK LVO specificity was calculated using a threshold that achieved matched sensitivity (corresponding JLK LVO thresholds: 0.07, 0.11, 0.42, and 0.82, respectively).

Supplementary Table 2. Sensitivity by occlusion site for JLK LVO and RAPID CTA

Location	Software	Threshold	Total	TP	FN	Sensitivity
ICA	JLK	0.5	25	23	2	0.92 (0.75–0.98)
	RAPID	<60%	25	23	2	0.92 (0.75–0.98)
MCA M1	JLK	0.5	18	18	0	1.00 (0.82–1.00)
	RAPID	<60%	18	17	1	0.94 (0.74–0.99)
MCA M2	JLK	0.5	10	3	7	0.30 (0.11–0.60)
	RAPID	<60%	10	4	6	0.40 (0.17–0.69)

TP=true positive, FN=false negative, ICA=internal carotid artery, MCA=middle cerebral artery

Supplementary Table 3. Sensitivity by laterality for JLK LVO and RAPID CTA

Laterality	Tool	Threshold	Total	TP	FN	Sensitivity
Left	JLK	0.5	27	22	5	0.82 (0.63–0.92)
	RAPID	<60%	27	24	3	0.89 (0.72–0.96)
Right	JLK	0.5	22	18	4	0.82 (0.62–0.93)
	RAPID	<60%	22	17	5	0.77 (0.57–0.90)
Bilateral	JLK	0.5	4	4	0	1.00 (0.51–1.00)
	RAPID	<60%	4	3	1	0.75 (0.30–0.95)

TP=true positive, FN=false negative

Supplementary Table 4. Analysis of false positive cases

No	FP Classification	Brief Description	Overlapped Case
1	C		
2	B-3	Vessel Extraction Error	
3	B-2	Previous coil embolization (A-com)	
4	A-1	Moderate stenosis	Yes
5	A-1	Partial occlusion with midline shift	
6	A-1	Near-total occlusion	
7	C	*	
8	A-1	Severe stenosis	
9	A-1	Moderate stenosis	
10	C		Yes
11	C	*	
12	C		
13	B-3	Vessel Extraction Error	
**No**	**FP Classification**	**Brief Description**	**Overlapped Case**
1	B-2	Previous aneurysm clipping	
2	B-3	Vessel Extraction Error	
3	A-1	Moderate stenosis	Yes
4	C		Yes
5	B-2	Previous coil embolization (ACA)	

FP=false positive, A-com=anterior communicating artery, ACA=anterior cerebral artery

* Posterior circulation occlusions (e.g., PCA, BA) were excluded from analysis, as both RAPID CTA and JLK LVO algorithms provide detection results for anterior circulation only.

Supplementary Table 5. Analysis of false negative cases

No	Location	FN Classification	Brief Description	Overlapped Case
1	Lt MCA-M2	A-2, B-1	Short-segmental occlusion with good collateral and preserved vessel density	Yes
2	Rt ICA	A-1	M2 superior occlusion with poor collateral	
3	Lt MCA-M2	A-1	M2 inferior occlusion, but relatively preserved vessel density	Yes
4	Both ICA	B-2	Chronic bilateral ICA occlusion. Reconstitution of bilateral M1 via basal collaterals	
5	Rt MCA-M1	A-2, B-1	Underlying intracranial atherosclerosis with progression. Short-segmental occlusion with good collateral	
6	Lt MCA-M2	A-1	Relatively small branch occlusion with moderate collateral	Yes
7	Rt MCA-M2	A-2, B-1	Tandem occlusion with ICA reconstitution and short-segmental occlusion with focal poor collateral	Yes
8	Rt MCA-M2	A-2, B-1	M2 inferior proximal segmental occlusion. Good collateral.	Yes
9	Rt MCA-M2	A-2, B-1	M2 inferior distal segmental occlusion. Good collateral.	Yes
**No**	**Location**	**FN Classification**	**Brief Description**	**Overlapped case**
1	Lt MCA-M2	A-2, B-1	Short-segmental occlusion with good collateral and preserved vessel density	Yes
2	Lt ICA	A-2, B-1	Left ICA and segmental M1 occlusion. Good collateral	
3	Lt ICA	A-1	Left ICA and M2 occlusion. Moderate collateral	
4	Lt MCA-M2	A-1	M2 inferior occlusion, but relatively preserved vessel density	Yes

5	Rt MCA-M2	A-1	Small M2 branch occlusion. Near-total occlusion in mid M2. Moderate collateral	
6	Lt MCA-M2	A-1	Relatively small branch occlusion with moderate collateral	Yes
7	Rt MCA-M2	A-2, B-1	Tandem occlusion with ICA reconstitution and short-segmental occlusion with focal poor collateral	Yes
8	Rt MCA-M2	A-2, B-1	M2 inferior proximal segmental occlusion. Good collateral.	Yes
9	Rt MCA-M2	A-2, B-1	M2 inferior distal segmental occlusion. Good collateral.	Yes

Lt=left, Rt=right, FN=false negative, ICA=internal carotid artery; MCA=middle cerebral artery

Supplementary Figure 1. Receiver operating characteristic curves of JLK LVO and RAPID CTA

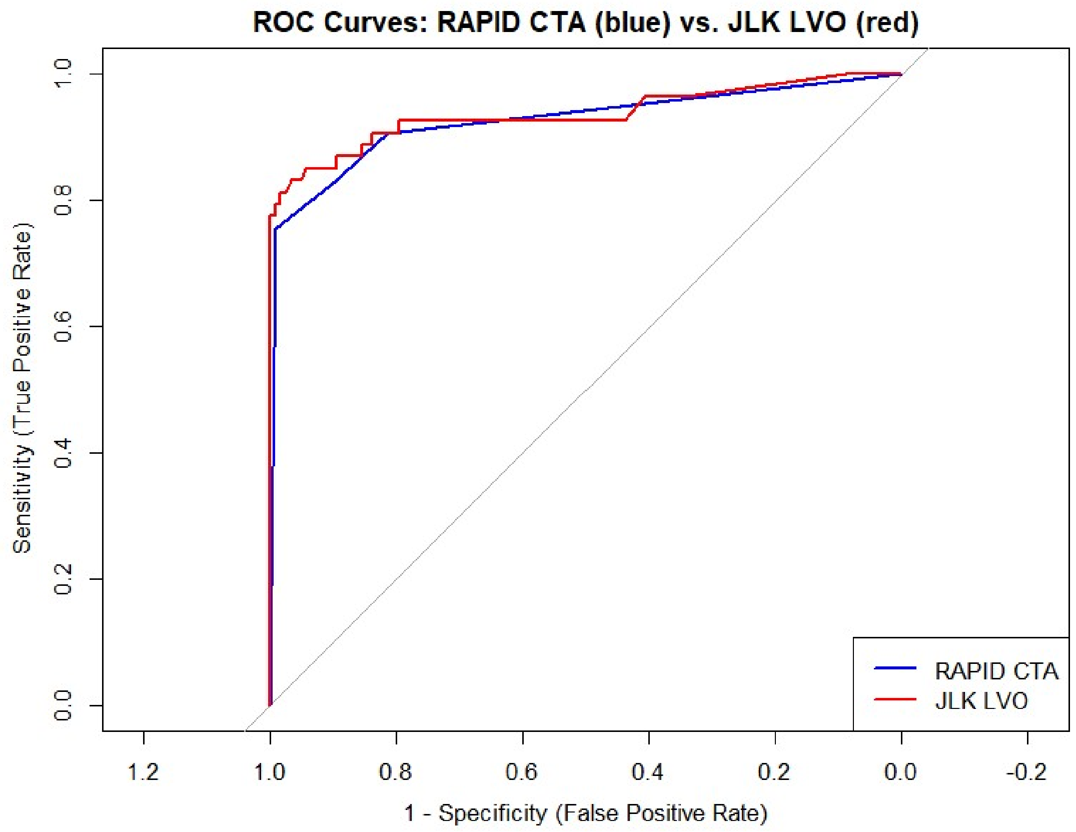

ROC=receiver operating characteristic

Supplementary Figure 2. Confusion Matrices of JLK LVO and RAPID CTA compared with neuroradiologists' interpretation as ground truth

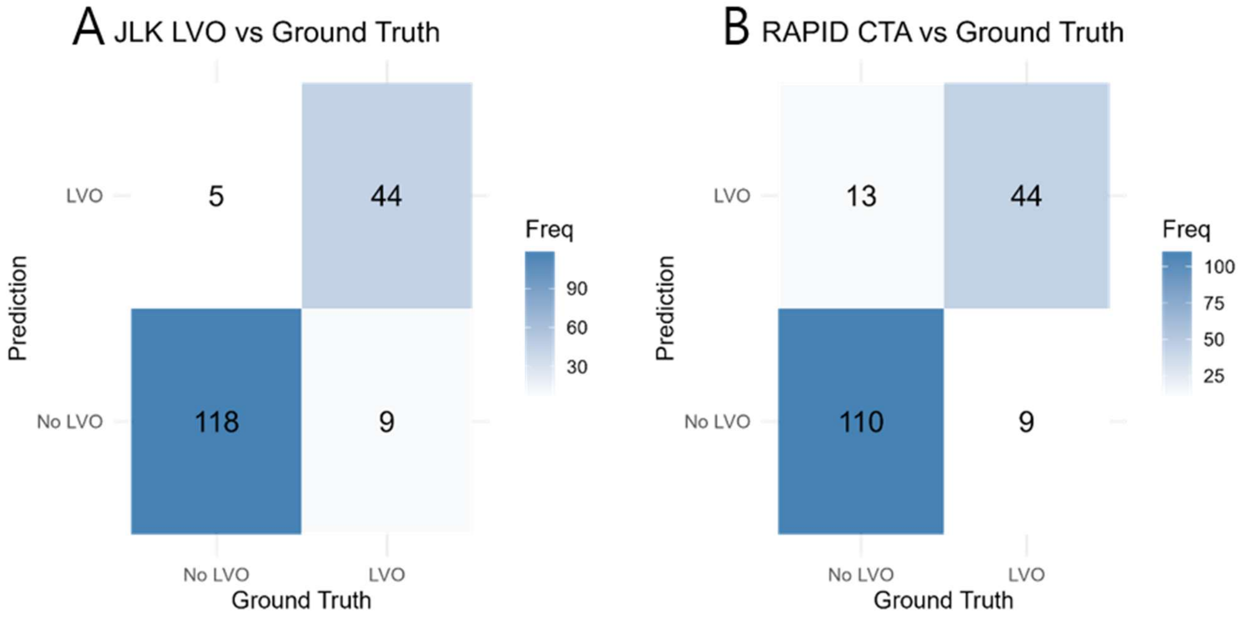

LVO=large vessel occlusion
